# The Development and Characterization of a Cotton–Chitosan Composite for Lead Removal from Water

**DOI:** 10.3390/polym13132066

**Published:** 2021-06-23

**Authors:** Diana Alonso-Segura, Luis Hernández-García, Jorge Menchaca-Arredondo, Mario Sánchez, Belén Chamorro-Garza, Raquel Garza-Hernández

**Affiliations:** 1 Biotechnology Engineering Division, Universidad Tecnológica de Corregidora, Carretera Estatal 413, Sta. Barbara Km. 11.2, Coroneo 76900, Mexico; 2 Instituto Tecnológico de Nuevo León, Av. de la Alianza 507, Parque de Investigación e Innovación Tecnológica 66628, Mexico; jose.luis.hernandez@itnl.edu.mx (L.H.-G.); bchgarza@gmail.com (B.C.-G.); 3 Facultad de Ciencias Físico Matemáticas, Universidad Autónoma de Nuevo León, Av. Universidad s/n, San Nicolás de los Garza 66455, Mexico; jorge.menchacarr@uanl.edu.mx; 4 Centro de Investigación en Materiales Avanzados, Alianza Norte 202, Parque de Investigación e Innovación Tecnológica 66628, Mexico; mario.sanchez@cimav.edu.mx (M.S.); raquel.garza@cimav.edu.mx (R.G.-H.)

**Keywords:** chitosan, cotton fiber, composite, lead

## Abstract

Heavy metals in water are a serious environmental problem due to their accumulation and toxicity; there are several processes we can use to address this issue, but adsorption is the most popular due to its simplicity and efficiency. Polysaccharides such as cellulose have received attention as adsorbents for heavy metals, and cotton–chitosan composites (CCs) were developed here with nontoxic reagents such as carboxylic acids as crosslinkers and NaH_2_PO_4_ as a catalyst to achieve chitosan covalent crosslinkage into oxidized cotton textiles with H_2_O_2_. The composites were characterized by fourier-transform infrared spectroscopy (FTIR), elemental analysis (EA), X-ray photoelectron spectroscopy (XPS), atomic-force and scanning electron microscopy (AFM and SEM), and tensile strength; the adsorption of lead ions (Pb) was evaluated with cotton–chitosan composites and quantified by microwave plasma atomic emission spectroscopy (MP-AES). The composites showed a maximum incorporation of chitosan of 27.62 mg per gram of cotton textile. A tensile strength analysis of the composite showed a Young’s modulus approximately 1 MPa higher than that of cotton textile. The adsorption of lead ions with composites in an aqueous solution at pH 5 and 25 °C was circa 74% after 6 h of contact, as determined by MP-AES. This work is an approach to demonstrate the potential of these polysaccharides, modified by “green” procedures to remove pollutants from water.

## 1. Introduction

Heavy metals in water are a severe environmental problem that directly affects human health even at very low concentrations; most of them are considered persistent, nonbiodegradable, and toxic, and must be removed; hence, an enormous amount of research into how to clean water at a lower cost and with less energy is needed. Many methods have been used to remove metals from water, such as ion exchange, chemical precipitation, filtration, electrolytic processes, solvent extraction, adsorption, and biosorption [1,2,3,4,5]. Adsorption is one of the most popular methods due to its simplicity, low cost, efficiency, the regeneration capacity of the adsorbent, no sludge generation, and simple recovery; however, some adsorption mechanisms are still not fully understood [3,6,7,8]. Polysaccharides such as cellulose, cellulose derivates, cotton textiles, and chitosan have received significant attention over the last two decades because they are convenient for heavy metal adsorption and are environmentally friendly materials [1,3,5,9,10,11,12,13,14,15].

Cellulose is easily obtained from nature; it is hydrophilic and has good reaction properties, so is a very good option to use as an adsorbent, or it can be functionalized to enhance its adsorption performance [5,6,11,12]. In this matter, cellulose extracted from jute fiber was modified with methyl acrylate/acrylonitrile monomers by a free radical method to obtained poly(methyl acrylate)-grafted cellulose and/or poly(acrylonitrile)-grafted cellulose and used for the removal of heavy metal ions from wastewater [1]; or for instance, Yu et al. designed a hyperbranched polyamide and synthesized a dialdehyde cellulose to be crosslinked together by a reductive amination. The adsorption capacity of Cu(II) of this functionalized cellulose was 137 mg/g due to the increased number of active sites on the developed material [13].

On the other hand, chitosan, the deacetylated form of the polysaccharide chitin, mainly found in the exoskeleton of crustaceans, is versatile, nontoxic, and has a good adsorption capacity due to the presence of amino and hydroxyl groups in the polymer matrix that act as active sites [10,11,12]. Nonetheless, chitosan’s stability in acidic environments limits its applications, as do its low surface area and poor mechanical and thermal properties; therefore, crosslinking processes to improve it have been studied [10,11,12,14,15,16]. The chemical modification of chitosan or its chemical fixation by covalent crosslinking into cellulose or other polymer matrices may improve the endurance and efficiency of the resultant composite, as reported by Yang et al., who crosslinked carboxymethylated chitosan and nanocrystalline cellulose through a Schiff base reaction to obtain a reusable adsorbent over a wide pH range [15].

The chemical fixation of chitosan and its covalent crosslinkage may involve toxic reagents that have several constraints such as the mechanical properties of the matrix, fiber degradation, and above all, the release of toxic and irritant compounds during the elaboration process and throughout storage [17,18]. Polycarboxylic acids have been used as an alternative to poisonous crosslinkers [14,19,20,21]. Hence, the use of citric acid and low-toxicity oxidizing agents, such as potassium permanganate, sodium hypophosphite, or even a food-grade reagent such as monosodium phosphate, has been shown to promote adequate chemical fixation between chitosan and cellulose [20,21,22,23].

Hydrogen peroxide (H_2_O_2_), an environmentally friendly reagent, has been used in high concentrations (above 1 M) to modify the crystalline structures of polysaccharides, changing their physical and chemical properties through the formation of free radicals, which attack the glycosidic linkages of polysaccharides [24,25,26].

In this work, hydrogen peroxide was used for the pretreatment of cotton textiles by oxidation; citric acid (CA) and butanetetracarboxylic acid (BTCA) were used as crosslinkers, with sodium phosphate as a catalyst, to attain chitosan covalent crosslinkage into cotton textiles. Hence, environmentally friendly composites were developed herein, based on an easy, cheap, and low-energy method, to be used for heavy metal adsorption in water. In this case, we evaluated lead adsorption at trace levels in water, as lead has been identified as one of the toxic elements that, in the aqueous phase, accumulate across the food chain and can cause serious diseases such as renal failure, brain damage, and cancer [14]. Therefore, this work is an attempt to demonstrate the potential of “green” composites based on chitosan–cotton textiles that help to remove traces of toxic elements, can be reused and eventually biodegrade due to their natural composition.

## 2. Materials and Methods

Chitosan (deacetylation degree ≥ 75%) and butanetetracarboxylic acid (BTCA) were purchased from Sigma-Aldrich (San Luis, MO, USA); cotton textiles were purchased from La Parisina Fabric Store (Mexico City, Mexico); H_2_O_2_ at 35% *v*/*v* food grade was from Je Chemistry (Monterrey, Mexico); citric acid (CA) and sodium phosphate (NaH_2_PO_4_) were from Fermont (Monterrey, Mexico); and acetic acid and nitric acid (HNO_3_) were purchased from JT Baker (Allentown, PA, USA). All reagents were used as supplied. The UV light lamp UVGL-58 was from Ultra-Violet Products (Jena, Germany).

### 2.1. Composite Development

Cotton–chitosan composites (CCs) were prepared by the pretreatment of cotton textiles with H_2_O_2_, followed by a crosslinkage reaction through citric acid or butanetetracarboxylic acid with chitosan and NaH_2_PO_4_ as a catalyst. All the composites developed herein are summarized in Table 1 and described below: aqueous solutions of chitosan (1.5% *w*/*v*), one with 4% *w*/*v* of citric acid (CA), and another with 4% *w*/*v* of butanetetracarboxylic acid (BTCA), were prepared. Both polycarboxylic acids were used as solvents for chitosan and as crosslinkers for the composites. Pieces of cotton textile of approximately 15 cm^2^ each were pretreated and submerged in H_2_O_2_ for 40 min; after that, excess peroxide was removed with distilled water and the samples were dried overnight.

Next, the pretreated cotton textile pieces (with hydrogen peroxide) were immersed into a chitosan aqueous solution with CA or BTCA. After that, 2.3% *w*/*v* of NaH_2_PO_4_ was added to the mixture, which was heated to 70 °C for 5 min in order to obtain the composites; these were then removed from the solution and dry-cured in a thermal oven at 130 °C for 3 min, as reported before [21]. CCs were dried at room temperature, washed with commercial ionic soap, rinsed with distilled water, followed by a wash of acetic acid solution (0.1 M) in order to remove noncrosslinked chitosan from the composites, rinsed with distilled water several times until the acidic smell was gone, dried overnight, and weighed prior to analyses.

### 2.2. Composites’ Characterization

An elemental analysis to quantify the nitrogen incorporated into the composites was conducted on a CHN/O Perkin Elmer 2400 Series II (Shelton, CT, USA).

Fourier transformed infrared analysis (FT-IR) of the composites, as well as of the cotton textiles, was conducted on a Nicolet iS10 (Waltham, MA, USA) with an attenuated total reflectance (ATR) accessory with a diamond tip. Each sample was scanned 64 times at a wavelength of 4000–400 nm.

Tensile testing was carried out on a MTS TestSuite**™** TWElite (Eden Prairie, MN, USA) at a speed of 4.23 mm/s; composites and cotton textiles were cut into strips of 100 ± 1.5 × 15.09 ± 1.24 mm with an average thickness of 0.4 ± 0.25 mm, and samples were tested by triplicate to obtain the average value.

The composites and cotton textiles were observed using a scanning electron microscope Nova NanoSEM200 (FEI, Hillsboro, OR, USA) at low vacuum, using a low vacuum detector (LV), and a NT-MDT NTEGRA Prima AFM at room temperature, with a RTESPA probe (Bruker, Mexico City, Mexico) of spring constant k = 40 N/m in intermittent contact mode. Images of height, deflection, and phase were obtained; 100 × 100 and 20 × 20 µm^2^ image sizes were captured systematically for each sample in the three different regions. They were analyzed with Nova 3.1 to obtain the average roughness and morphological aspect of the composites.

### 2.3. Lead, Pb(II), Adsorption–Desorption Determination

Adsorption and desorption experiments related to lead removal from water were determined twice in batches. The Pb ion solutions were analyzed with an Agilent MP-AES 4200 microwave plasma-atomic emission spectrometer (Santa Clara, CA, USA), at a wavelength of 368.34 nm and a nebulizer pressure of 0.95 L/min. The adsorption capacity *q_e_* (mg/g), % adsorption of Pb, and % desorption were calculated according to the following Equations (1)–(3) [1,12,14]:

(1) $$q_{e}=\frac{\left(C_{0}-C_{e}\right)V}{m}$$

(2) $$\%Adsorption=\frac{\left(C_{0}-C_{e}\right)}{C_{0}}100$$

(3) $$\;\%\;\mathrm{Desorption}=\frac{C_{\mathrm{ion}\;\mathrm{desorbed}\;\mathrm{by}\;\mathrm{eluent}}}{C_{\mathrm{initial}\;\mathrm{of}\;\mathrm{ion}\;\mathrm{adsorbed}\;\mathrm{on}\;\mathrm{adsorbent}}}100$$

where *C_0_* and *C_e_* (mg/L) are the initial and equilibrium concentrations of lead, *V* (L) is the volume of solution, and *m* (g) is the mass of composite used as adsorbent.

Lead adsorption by CCs was evaluated, at pH 5, with Pb in HNO_3_ at 3% *v*/*v* and Pb in H_2_O_2_ at 4% *v*/*v* at 25 °C; the pH was adjusted with NaOH 0.1 N or with HNO_3_ 0.1 M. Then, 250 mg of dried composites were weighed and submerged into 100 mL of Pb solution with an initial concentration, *C_0_*, of 40 mg/L for 6 h at room temperature. In order to analyze the metal ion concentration adsorbed, aliquots were taken every 2 h.

To elute adsorbed lead ions, composites from adsorption determinations were submerged in 100 mL of nitric acid (HNO_3_) solution, 0.1 M, for 6 h at room temperature and agitation (75 rpm), every 2 h aliquots were taken and analyzed with MP-AES.

### 2.4. X-ray Photoelectron Spectroscopy (XPS)

X-ray photoelectron spectroscopy was used to determine the surface composition and chemical states of the composites before and after the adsorption determinations. The measurements were performed in an X-ray spectrometer (Thermo Scientific Escalab 250 Xi; Santa Clara, CA, USA). The photoelectrons were generated with monochromatic Al Kα (1486.7 eV) as the X-ray source with a line width of 0.20 eV. The ultra-high vacuum analysis chamber was kept at a base pressure of <4 × 10^−8^ Pa. The electrons were detected using a hemispherical analyzer with a pass energy of 20 eV. The relevant core levels C 1s, N 1*s*, O 1*s*, and Pb 4*f* were considered to quantify the elemental atomic contents, using the sensitivity factors from the instrument database. Corrections to the shift in the binding energy were made by fixing the energy of the C1*s* peak, from adventitious carbon in the films, at 284.8 eV. The fitting procedure was an iterative process whereby all the peaks were fitted using the AAnalyzer^®^ (CINVESTAV-Queretaro, Santiago de Querétaro, Mexico) peak fitting software.

## 3. Results

### 3.1. Composites’ Characterization

#### 3.1.1. Elemental Analysis

The results of the elemental analysis indicated that the composites, after being subjected to several washes, presented a high nitrogen content; this was attributed to the crosslinked chitosan into cotton textiles using either citric acid or butanetetracarboxylic acid; the nitrogen determinations are presented in Table 2.

The composites where H_2_O_2_ was used with CA or with BTCA as crosslinkers displayed the highest chitosan incorporation of 20.13 mg/g_composite_ and 27.62 mg/g_composite_, composites 3 and 4, respectively. When a catalyst (NaH₂PO₄) was not used, the chitosan incorporation was circa 13.56 mg/g_composite_; when CA was the crosslinker and 22.6 mg/g_composite_ with BTCA (composites 1 and 2, Table 2), there was almost 5 mg/g_composite_ less than when the catalyst was used.

#### 3.1.2. Fourier Transform Infrared Spectra

**Figure 1:**
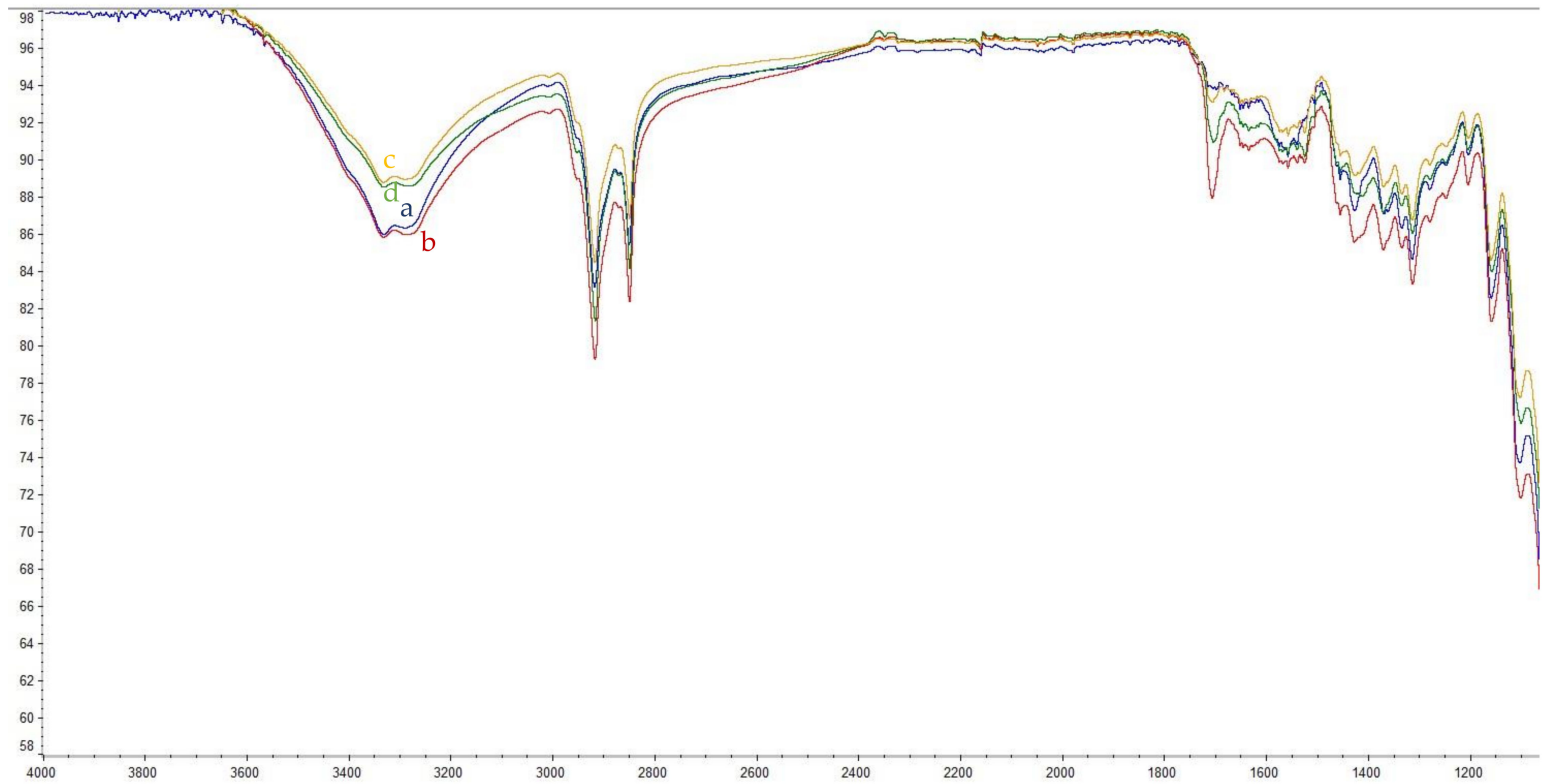

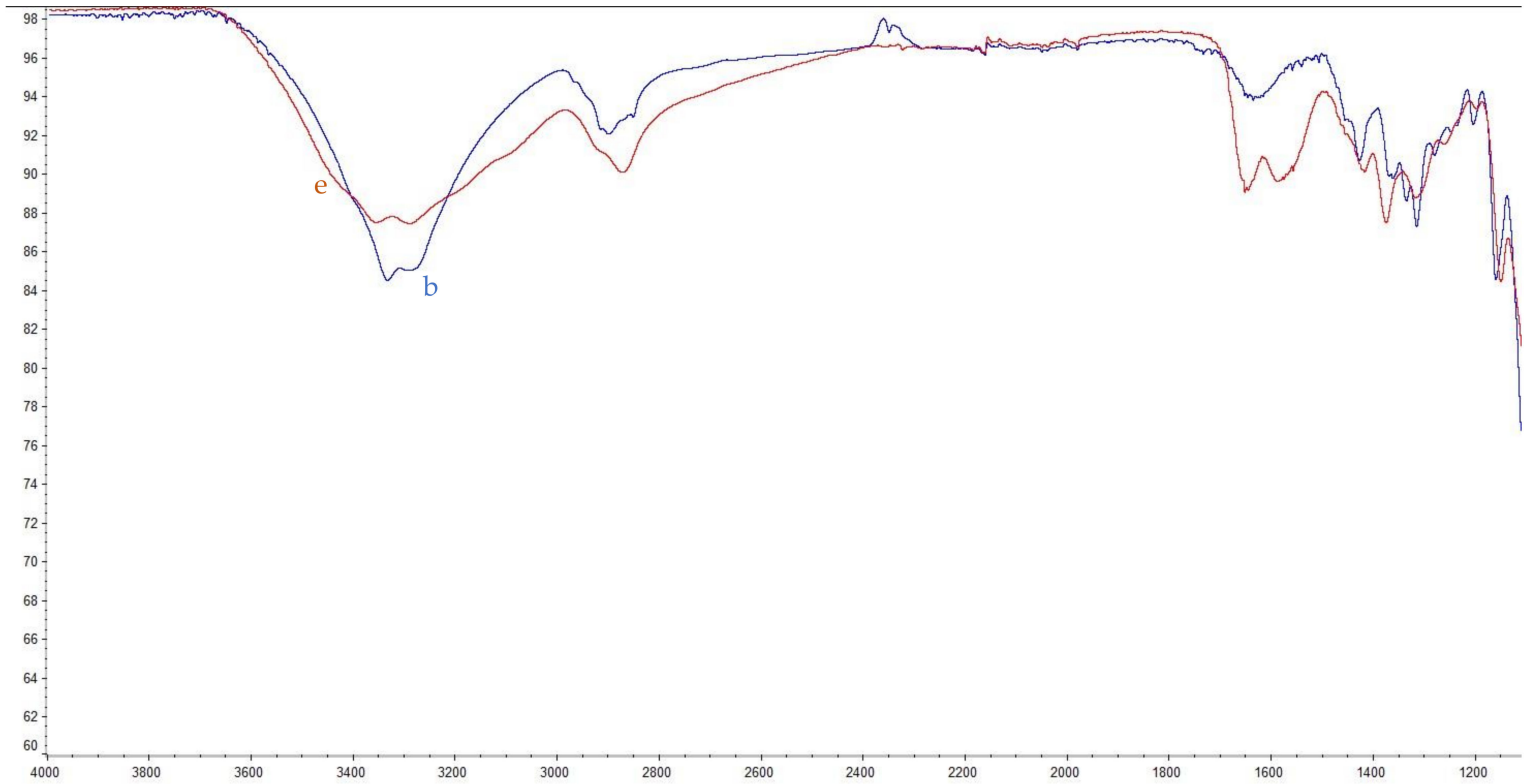
FTIR spectra: (**a**) composite 4, navy blue; (**b**) composite 2, red; (**c**) composite 3, yellow; (**d**) composite 1, green; (**e**) chitosan, orange; (**f**) cotton textile, blue.

The FT-IR spectra of composites 3 and 4 show those bands related to the primary amine group that are slightly visible at 1560 cm^−1^; the peaks at 2927 and 2839 cm^−1^ are attributed to C–H stretching of the alkyl group of cellulose as well as from the chitosan (Figure 1). Also, the presence of a C=O ester stretching band at 1705 cm^−1^ is more intense for composite 3 when CA and hydrogen peroxide were used for its elaboration, which indicates that the covalent attachment of chitosan into cotton textiles was by an ester bond formation, as reported elsewhere [21].

#### 3.1.3. Tensile Strength

**Figure 2:**
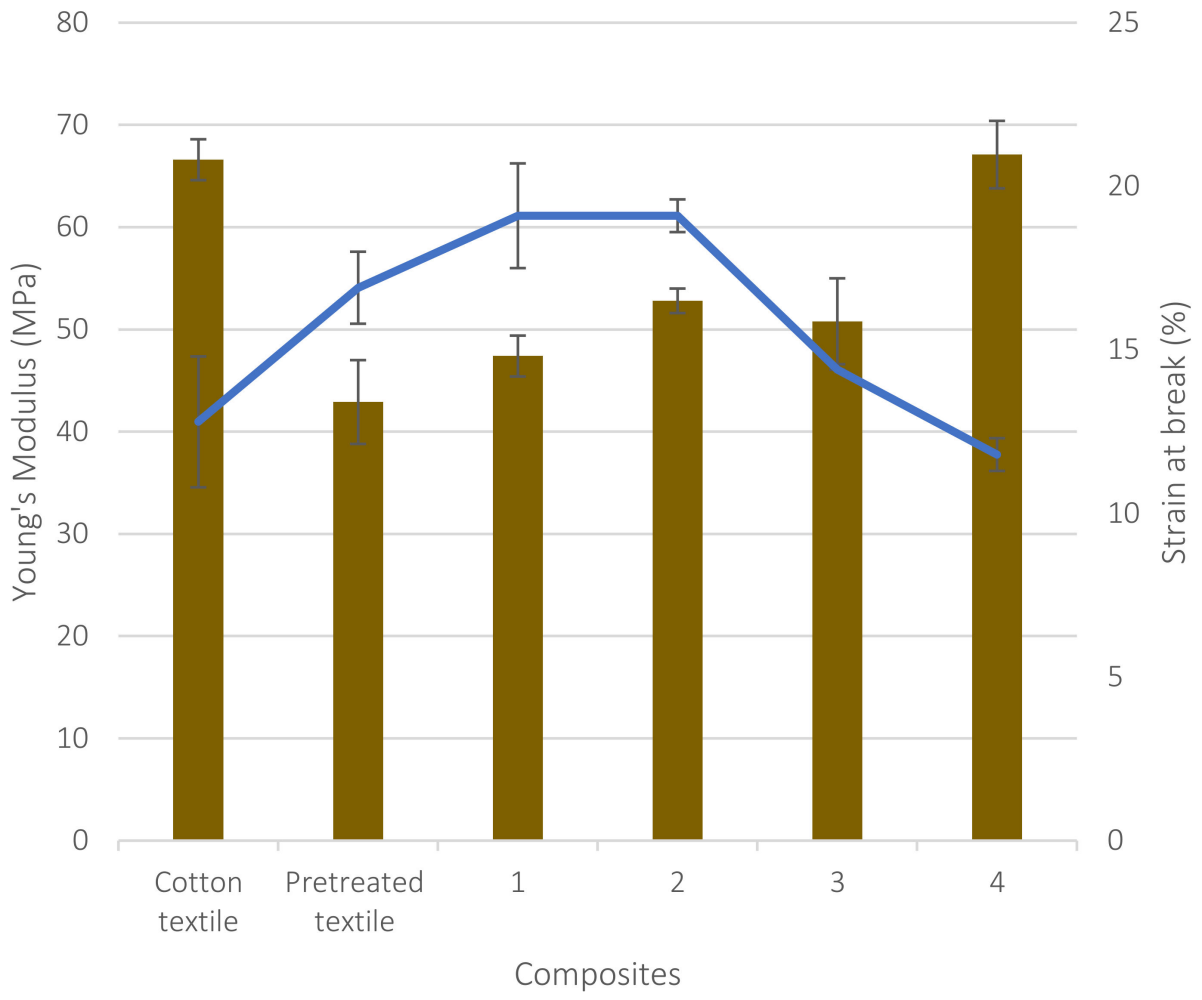
Average tensile strength and strain at the breaking point of cotton textiles, pretreated textiles with H_2_O_2_, and composites.

The tensile strength properties of the composites developed in this work show that the use of H_2_O_2_ as a pretreatment for cotton textiles reduces its elasticity, but with chitosan crosslinkage, there is an increase in the tensile strength observed. The stronger composite (composite 4), the one with the higher chitosan concentration crosslinked, has a modulus of 67.1 ± 3.3 MPa, approximately 1 MPa more than the cotton textile used herein. The relationship between the Young’s modulus and the strain at the breaking point of composites, as well as from the cotton textiles, are shown in Figure 2, with composite 4 being the one with the smallest strain at the breaking point, and hence the composite with the highest modulus.

#### 3.1.4. Atomic Force and Scanning Electron Microscopy

**Figure 3:**
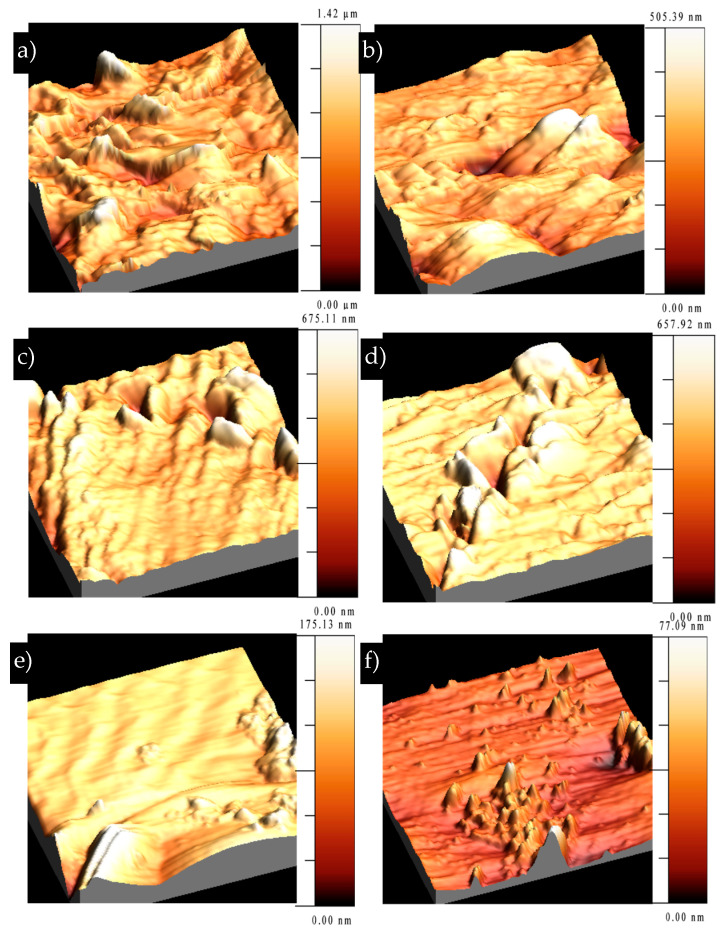
Representative AFM images of (**a**) cotton textile, (**b**) pretreated textile with H_2_O_2_, (**c**) composite 1, (**d**) composite 2, (**e**) composite 3, and (**f**) composite 4.

The topographical examination of composites by AFM revealed changes in the textile surface, as shown in Figure 3; these changes are related to chitosan crosslinkage. Cotton textiles presented a rough surface with an average height of circa 1.43 μm, and these values changed with chitosan incorporation, so the greater the chitosan concentration determined in composites, the smoother the surface analyzed. Composite 4, which showed almost 27 mg of chitosan for each gram of composite (Table 2), displayed the smoothest surface, with an average height of 77 nm.

**Figure 4:**
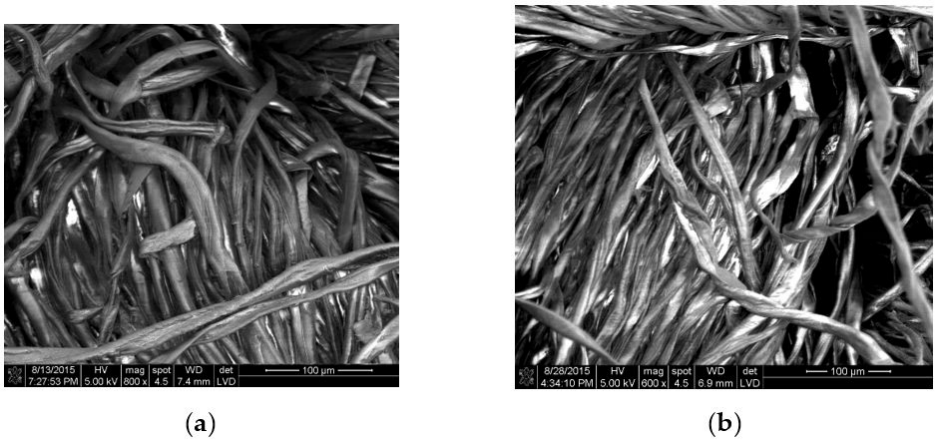

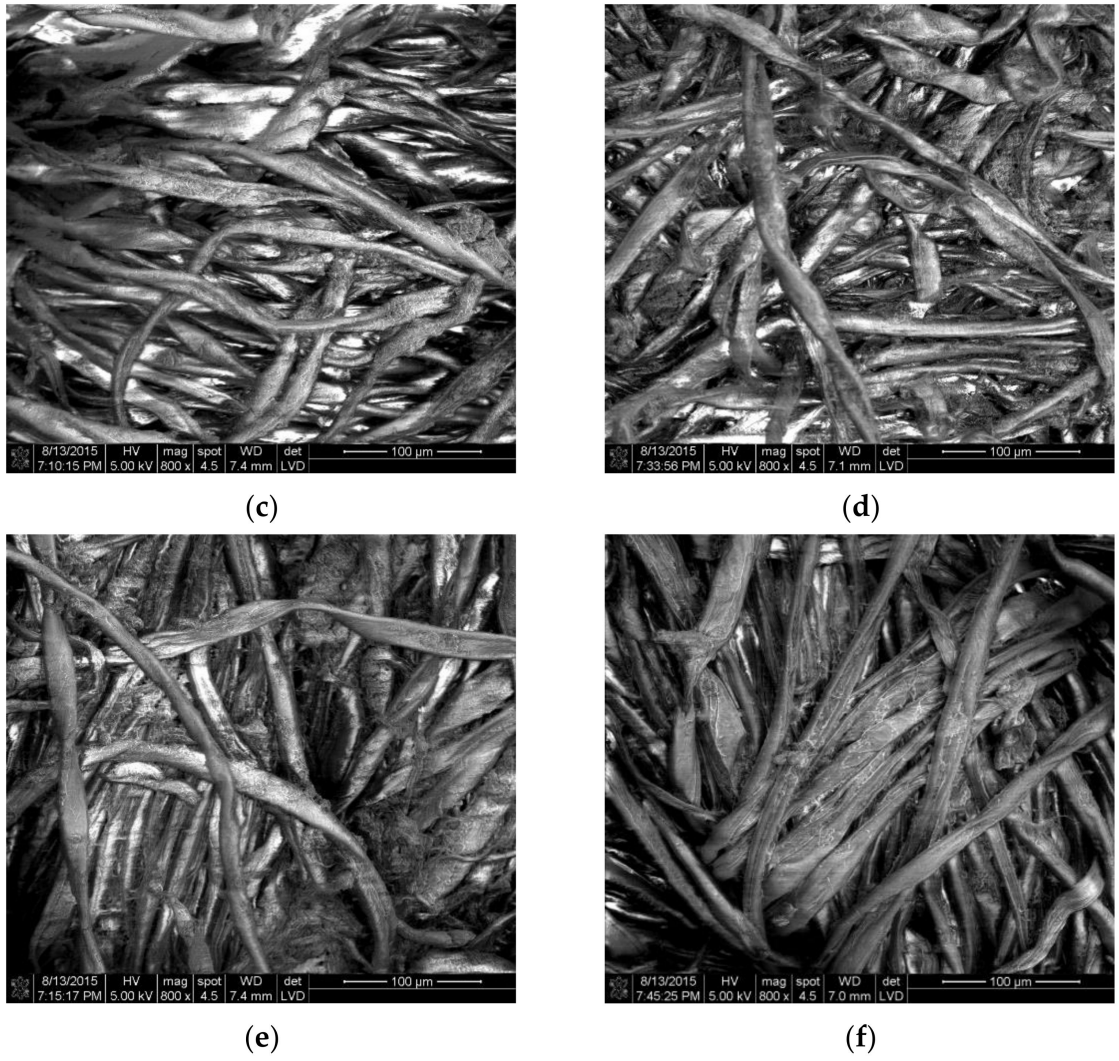
SEM images of (**a**) cotton textile, (**b**) pretreated textile with H_2_O_2_, (**c**) composite 1, (**d**) composite 2, (**e**) composite 3, and (**f**) composite 4.

In SEM micrographs of composites, pristine cotton textile and cotton textile oxidized with hydrogen peroxide are shown in Figure 4. It can be observed that the oxidized textile (Figure 4b) look similar to the pristine cotton textile (Figure 4a). Changes in morphology can be observed in the developed composites (Figure 4c–f). Composites presented extra material like lumps, making the surface heterogeneous as well as less even, being more noticeable in micrographs that correspond to composite 3 and 4 (Figure 4e, 4f), which demonstrate chitosan crosslinkage into cotton textile.

### 3.2. Lead Adsorption from Water

**Figure 5:**
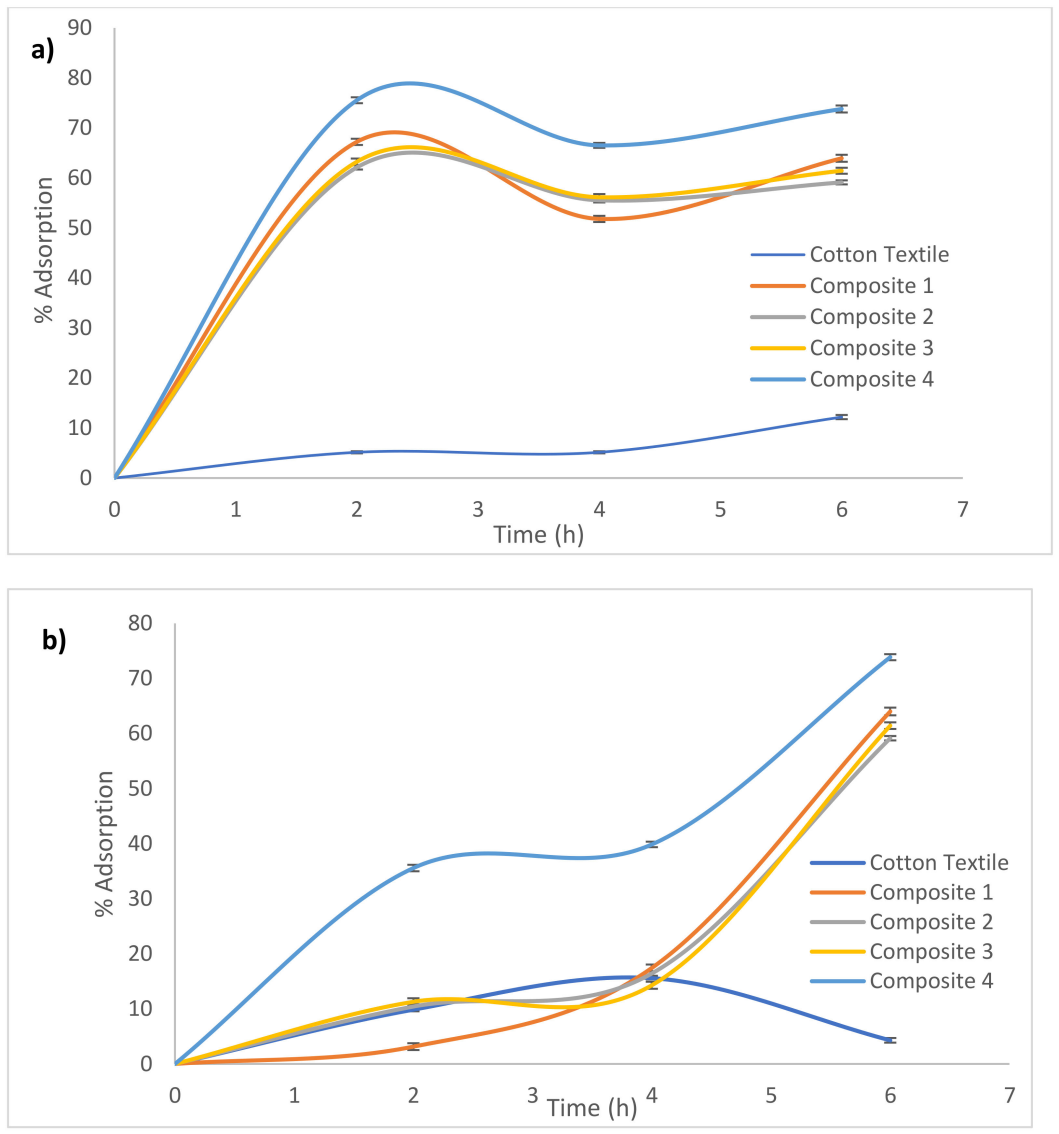
Lead adsorption percentage of composites and cotton textiles in (**a**) PbO solution or (**b**) Pb(NO_3_)_2_ solution, both at pH 5.

The best adsorption capacity is carried out in aqueous solution, as reported in several works at pH 5 [1,12,14], here, it was 75.54% during the first 2 h with composite 4. The higher the chitosan content in the composite, the faster the adsorption, reaching almost 74% during the first 2 h, with no increases after 6 h (composite 4). This occurred when PbO was used, but not when Pb(NO_3_)_2_ was the lead ion source (Figure 5); during the first 2 h the adsorption percentage was 35%, with the highest adsorption percentage being 74% for composite 4 after 6 h.

**Figure 6:**
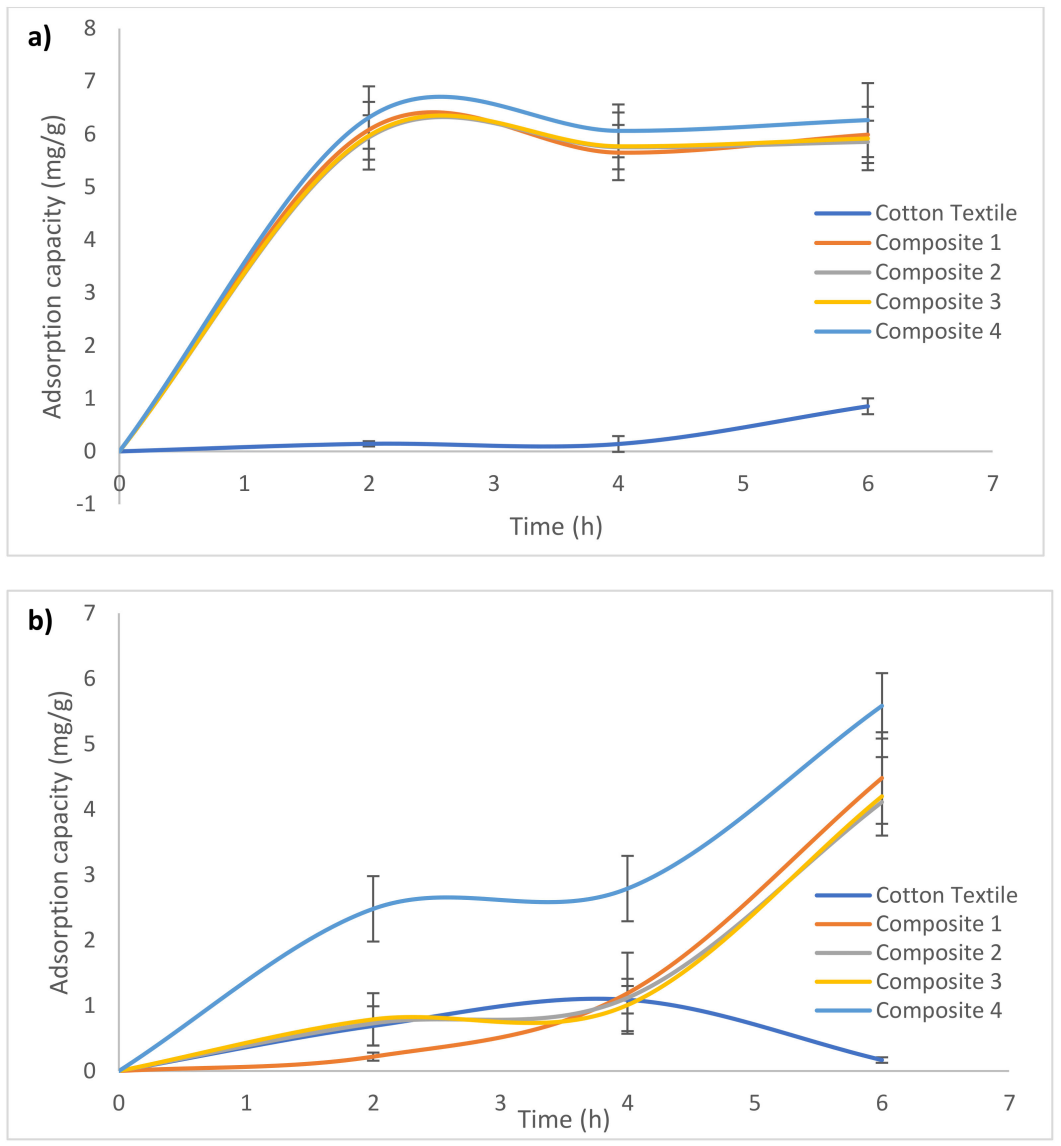
Adsorption capacity of composites and cotton textiles in (**a**) PbO solution or (**b**) Pb(NO_3_)_2_ solution, both at pH 5.

In Figure 6, the amount of Pb(II) adsorbed (*q*_e_) is presented. The PbO solution adsorbed lead ions at circa 6 mg/g of composite with all composites developed here after the first 2 h of contact, almost 7 times higher than with a pristine cotton textile. On the other hand, with the solution of Pb(NO_3_)_2_, after 2 h only composite 4 adsorbed 2 mg/g, almost 3 times more than cotton textile, and after 6 h most of the composites (1, 2, and 3) adsorbed almost 5 times more; composite 4 adsorbed the highest concentration of lead ions.

### 3.3. Lead Desorption from Composites

**Scheme 1:**
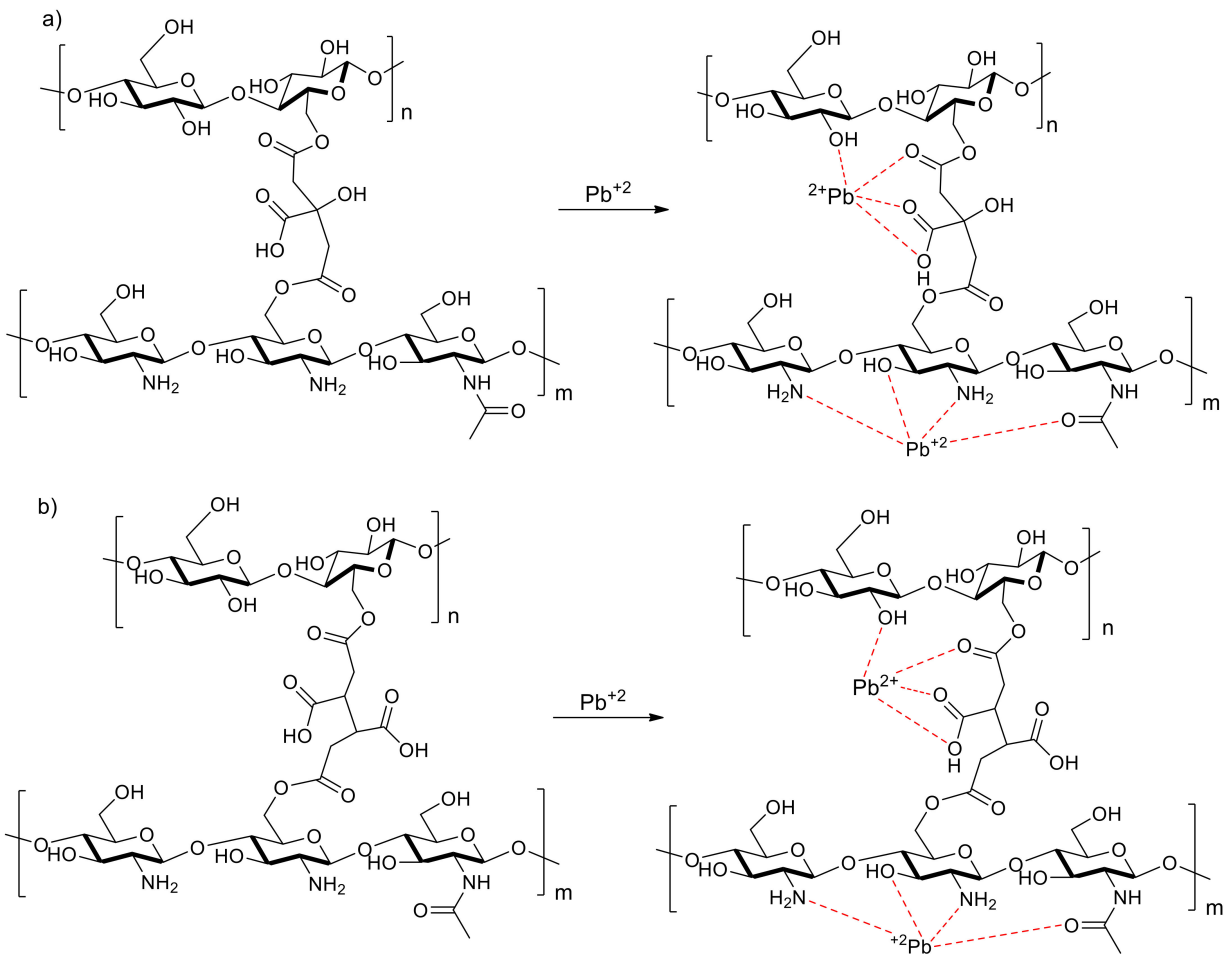
Proposed crosslinkage of cotton textile–chitosan composites: (**a**) citric acid as a crosslinker and composite interaction with lead; (**b**) butanetetracarboxylic acid as a crosslinker and composites’ interaction with lead.

**Figure 7:**
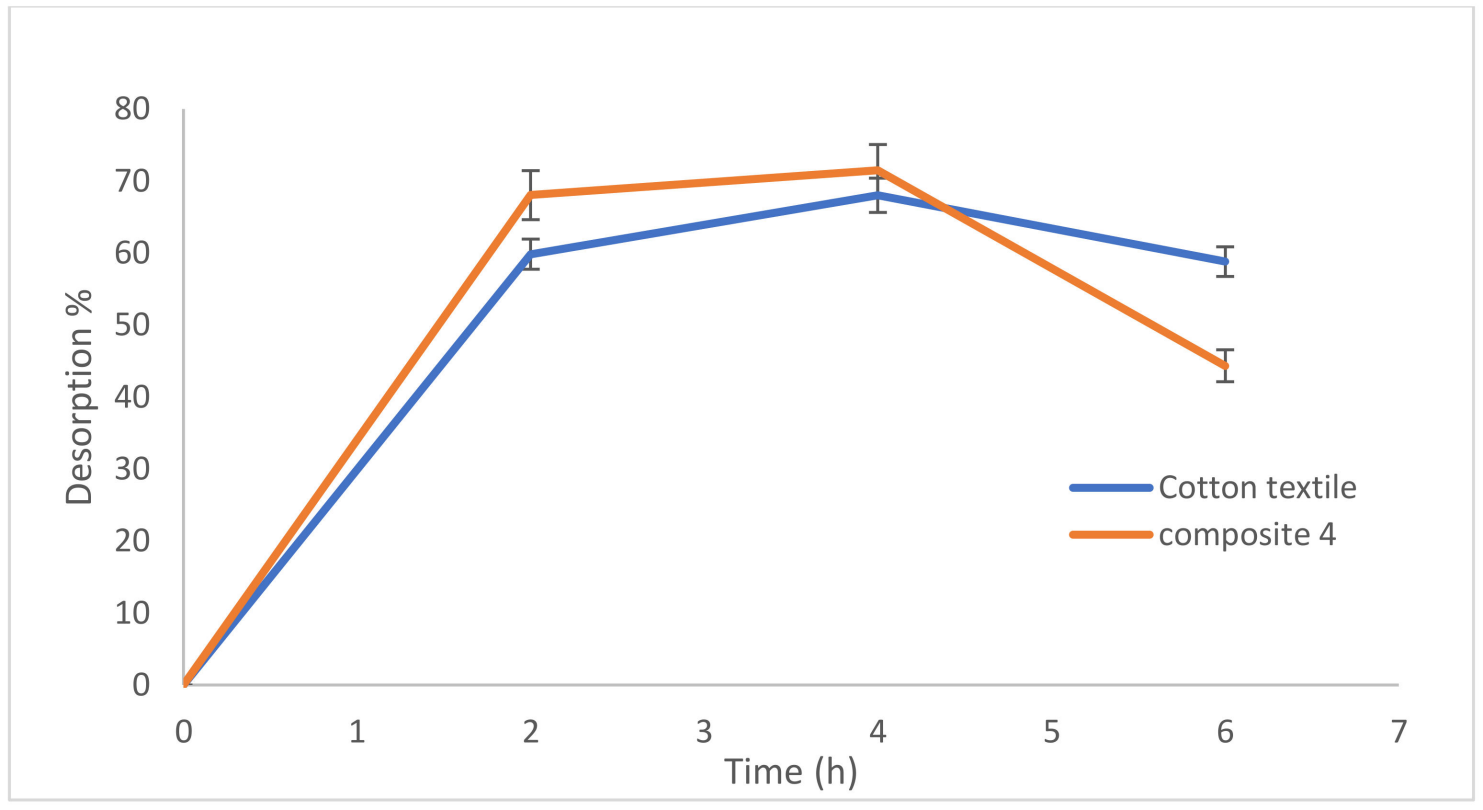
Desorption % of lead ions with composite 4 and cotton textile in nitric acid solution.

Desorption determination was carried out with an acid aqueous solution, where the lead ion of the complexes formed between CCs and lead, proposed in Scheme 1, makes a bond with the NO_3_^-^ and is eluted out from the CCs, desorbing almost 71.5% after 2 h from composite 4. A plateau was observed at 4 h, and 6% more lead was desorbed than in a cotton textile after the same time; however, after 6 h, composite 4 adsorbed the lead ions over again. Figure 7 demonstrates the interactions between composite moieties and lead ions presented in Scheme 1.

In the macroscopic structure of the developed composites, it is possible to observe different groups with free electron pairs, in which the metallic ions can have a stereoelectronic interaction. Given the number, geometry, and distance of the hydroxyl groups present in cellulose and chitosan, these give the biopolymer an attractive capacity as a lead adsorbent. Crosslinking agents such as BTCA have sp3 hybridized carbons that provide greater flexibility to the carbonyl groups to trap metal ions. On the other hand, the amino groups of chitosan presumably interact more with metal ions (Scheme 1), because those amino groups behave as a softer base than oxygen according to Pearson’s theory [27], which agrees with the data obtained from the elemental analysis, where composite 4 has the highest amount of chitosan and the best adsorption capacity.

### 3.4. Surface Analysis by XPS

**Figure 8:**
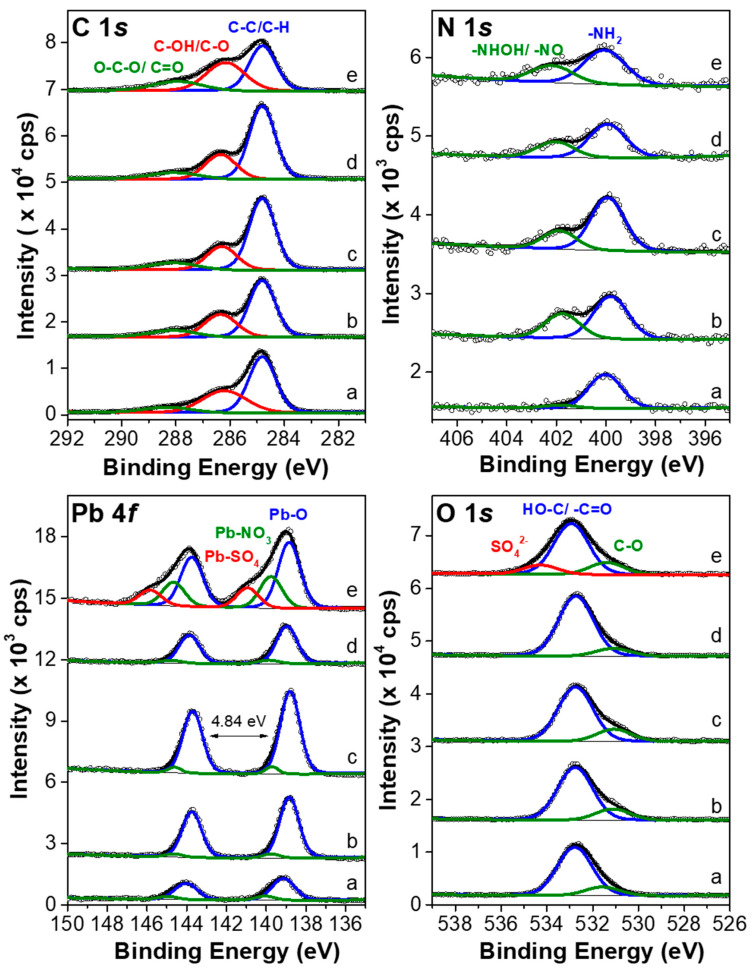
XPS high resolution spectra of core levels of C 1*s*, N 1*s*, O 1*s*, and Pb 4*f* for (**a**) cotton textile, (**b**) composite 1, (**c**) composite 3, (**d**) composite 2, and (**e**) composite 4.

The high-resolution spectra corresponding to the C 1*s*, O 1*s*, N 1*s*, and Pb 4*f* core energetic levels of the different composites after the adsorption of lead are shown in Figure 8. The main chemical difference between them is the extra carboxylic group for composites in which BTCA was used as crosslinker (composites 2 and 4), which can be used as a reactive site for the adsorption of lead. The C 1*s* spectra region is constituted of three main components. The first peak located at 284.8 eV is attributed to the C–H/C–C chemical bonds; moreover, the C–NH_2_ bond overlapped with this peak [28]. The second peak was centered at 286.3 eV and can be assigned to the C–OH, C–O, and C–N bonds [29]. The third peak at 288.0 eV corresponds to O–C–O/N–C=O, where components such as C=O or O=C–O can be overlapped by this peak [29,30]. The molecular structure of organic composites only exhibits one kind of nitrogen species (–NH_2_); nevertheless, two inflections were detected in the N 1*s* spectra. A single nitrogen signal was perceived in the pristine cotton textile at 399.9 eV, which is attributed to the nonprotonated amine from chitosan [31,32]. An extra component at 401.8 eV was found in the samples when oxidizing agents were used (H_2_O_2_). The oxidation of amines is relatively easy; thus, the component found at the higher binding energy was related to the –NHOH/–NO groups [33].

The detection of some metals at the surface by XPS can be difficult, especially when it is found with a low concentration in a matrix. The adsorption of Pb at the surface of the composites was possible because the “4*f*” orbital has a high sensitivity factor (22.74). The Pb 4*f* region is constituted of Pb 4*f*_7/2_ and Pb 4*f*_5/2_ core level peaks. The doublet peaks centered at 138.8 eV and 139.8 eV with a spin-orbit splitting of 4.84 eV are associated with the Pb–O and Pb–NO_3_ bonds, respectively [34]. An extra peak at high binding energy (140.9 eV) was observed only for composite 4. The spectra for O1s showed three peaks. The peak located at the lower binding energy (531.1 eV) was attributed to O–C bonding, which formed due to the adsorption of CO_2_ from the atmosphere. The O–Pb bond is complicated to distinguish because it has been found at 529–530 eV, which overlaps with the O–C peak [35,36]. The peak at 532.7 eV is assigned to the H-–O–C and O–C=O bonds [28,29,37,38], which corresponds to all carboxylic and hydroxy groups from the composites. As perceived in the Pb 4f region, there is a component at higher binding energy (534.2 eV) only for composite 4. This peak could be related to a super oxidized species or Pb–SO_4_ [35].

Table 3 shows the percentages of the relative atomic concentrations of all elements found in the samples. The atomic ratio O/C for the composites has been found to be around of 0.25–0.32. The atomic concentrations obtained by XPS revealed that crosslinking between cotton textile, chitosan, and BTCA or CA reagents occurred due to the O/C atomic ratio, which increased from 0.25 to 0.32. The adsorption of lead in all composites developed both BTCA and CA (composites 1–4) was higher than with pristine cotton textile. Particularly with those with BTCA, both composites 2 and 4 exhibited the highest lead content, proving that the extra carboxyl group is helpful for metal adsorption.

## 4. Discussion

The chitosan content of composites, as determined by an elemental analysis, showed that the use of a catalyst and an oxidizing agent (H_2_O_2_) favors chitosan covalent crosslinkage, mainly due to the surface modification of the cotton textile [21,24]. The H_2_O_2_ effect, besides discoloring the cotton textile, relies on the dissociation of perhydroxyl anion (HOO^–^), which predominantly occurs under alkaline conditions; hence, the action takes place when the nucleophile (HOO^–^) attacks the carbonyls and conjugated carbonyl groups that comprise the fiber [24,25]. There have been several reports of the chemical crosslinkage of chitosan within the free amino group; nevertheless, the crosslinker, chitosan concentration, catalyst, temperature, and reaction time are factors that affect the reaction site [5,9,11,12,15,16,17,18,21]. In this case, R-OH was more nucleophilic than R-NH_3_^+^, hence OH–C_6_ easily participated in nucleophilic reactions.

The adsorption capacity results showed that co-existing ions had adverse effects on the adsorption of lead over CCs. Thus, the presence of coexisting ions determined the adsorption capacity of CCs, which in this case was due to the crosslinkage with carboxylic acids; this can proceed in one of two ways: by chelation between nitrogen atoms of the NH_2_^–^ groups of chitosan and/or by electrostatic attraction between carboxyl groups of crosslinkers grafted into chitosan and cotton fiber. There are a few reports in which Pb species could be recognized at high binding energy by XPS analysis. However, carboxylic groups in the composites are responsible for promoting the adsorption of lead due to electronegativity, hence the binding energy found for Pb species by XPS suggests that the oxidation state of Pb in these composites was 2+. In addition, desorption determination confirmed that our composite formulation increased the number of active sites compared to the pristine cotton textile.

This work was an approach to demonstrate the potential ability of chitosan–cotton textile composites, developed with nontoxic reagents, to adsorb pollutants such as lead in water at trace levels. Once adsorbed, the pollutants can be concentrated in smaller quantities of acidic aqueous solution and the composites can be reused and eventually biodegraded.

It is worth mentioning that our research group is working with theoretical calculations to determine the affinity of several heavy metals for these composites, in addition to specifically determining the chemical modification of cellulose and chitosan by this kind of crosslinking processes, to reduce reagent consumption as well as waste generation.

## Figures and Tables

**Table 1:** Composites’ formulation.

Composite	H₂O₂ (35% *v*/*v*)	CA	BTCA	NaH₂PO₄
1	40 min	4% *w*/*v*	-	-
2	40 min	-	4% *w*/*v*	-
3	40 min	4% *w*/*v*	-	2.3% *w*/*v*
4	40 min	-	4% *w*/*v*	2.3% *w*/*v*

**Table 2:** Chitosan crosslinked into the composites developed herein.

Composite	mg_chitosan_ to g_composite_ *
1	13.56 ± 2.00
2	22.60 ± 1.81
3	20.13 ± 0.87
4	27.62 ± 5.03

* Results are the mean of three determinations and their standard deviations.

**Table 3:** Relative atomic concentration (%) for the different composites.

Orbital	C 1*s*			N 1*s*		O 1*s*			Pb 4*f*		
Composite	C–C/C–H	C–OH/C–O	O–C–O/C=O	NH_2_	–NHOH/–NO	O–C	OH–/O=C–	SO_4_^2−^	Pb–O	Pb–NO_3_	Pb–SO_4_
Cotton textile	43.6	27.7	7.1	1.2	0.0	16.9	3.0	0.0	0.3	0.1	0.0
1	43.7	21.4	8.8	1.5	0.9	18.8	4.0	0.0	0.8	0.1	0.0
3	48.8	18.5	8.4	1.6	0.6	17.2	3.9	0.0	1.0	0.1	0.0
2	49.0	19.5	8.3	1.1	0.5	18.6	2.4	0.0	0.5	0.0	0.0
4	31.4	29.2	12.1	1.3	0.7	16.6	4.0	3.0	0.9	0.4	0.3

## Data Availability

Not applicable.
